# Supplementary material for: Immunohistochemical Analysis of Mastocyte Inflammation: A Comparative Study of COPD Associated with Tobacco Smoking and Wood Smoke Exposure
Source: Biomedicines. 2025 Jun 30;13(7):1593. doi: 10.3390/biomedicines13071593 (PMC12292312; doi:10.3390/biomedicines13071593)
Supplement: Supplementary file 1 [file biomedicines-13-01593-s001.zip › biomedicines-3645993-supplementary.pdf]

Supplementary Material

Figure S1. Bland–Altman plot showing agreement between measurements of the total mast cell count.

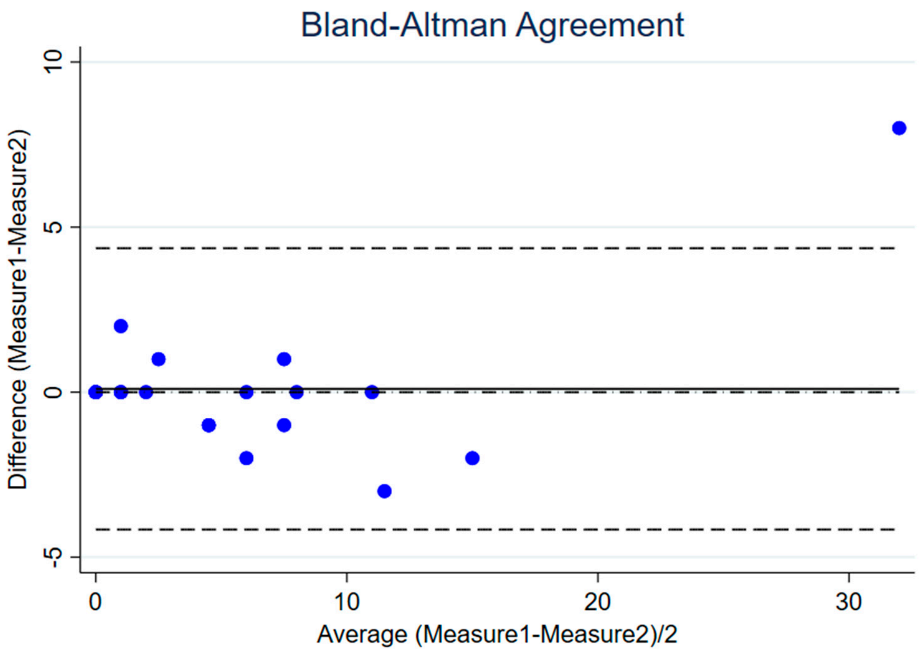

Mast cell count agreement by the same pathologist, Bias = 0.2 cells (95 % CI –0.9 to 1.0); LoA = –4.7 to +4.4 cells; Concordance = 0.96.
